# Chloroquine-Induced Psychosis: A Case Report

**DOI:** 10.7759/cureus.30498

**Published:** 2022-10-20

**Authors:** Hamzah E Chaudhry, Somieya Khan, Sidra Jamil, Tanveer Ahamad Shaik, Saad Ehsan Ullah, Anan Bseiso, Meenakshi Sathish, Faraz Saleem, Muhammad Abu Zar Ghaffari

**Affiliations:** 1 Medical School, California Institute of Behavioral Neurosciences & Psychology, Fairfield, USA; 2 Accident and Emergency, Glan Clwyd Hospital, Rhyl, GBR; 3 Internal Medicine, California Institute of Behavioral Neurosciences & Psychology, Fairfield, USA; 4 Cardiovascular Medicine, University of Louisville School of Medicine, Louisville, USA; 5 Internal Medicine, Shaikh Zayed Hospital, Lahore, PAK; 6 Surgery, Caribbean Medical University School of Medicine, Chicago, USA; 7 Internal Medicine, Akhtar Saeed Medical and Dental College, Lahore, PAK

**Keywords:** drug-induced psychosis, psychosis, chloroquine induced psychosis, chloroquine, anti-malarial therapy, anti-malarial

## Abstract

The use of antimalarial drugs for prophylaxis is a widespread practice while traveling to underdeveloped nations, particularly those with a high malaria prevalence. Chloroquine is still one of the most commonly recommended antimalarials, either alone or in combination with others, for prophylaxis. However, its increased use over the past few decades has been associated with many adverse effects, including headaches, dizziness, vomiting, and diarrhea, as well as neuropsychiatric symptoms such as psychosis. Here, we discuss the case of a 30-year-old Asian man who, after starting a 500-milligram (mg) prophylactic dosage of chloroquine per week, developed psychotic symptoms. This case highlights the need to use chloroquine and other antimalarials with care, especially when beginning as a prophylactic measure with the lowest suggested dosage.

## Introduction

Malaria and anti-malarial medications have both been associated with neuropsychiatric symptoms in patients. The psychiatric symptoms in patients who were given anti-malarial drugs, particularly chloroquine and mefloquine, include amnesia, insomnia, loss of focus, reduced attention, anxiety, depression, delirium, mania, and psychosis [1,2]. Antimalarials can result in adverse effects even when used as prophylaxis. Chloroquine, an antimalarial medication with a narrow therapeutic window and rapid onset of toxicity, has been linked to psychosis in a wide range of ages, including children [3]. Our case study is about a 30-year-old patient who had psychotic symptoms after using chloroquine as a malaria prophylactic for two to four weeks. This is one of the most uncommon adverse effects of chloroquine.

## Case presentation

A 30-year-old South Asian male from Denbighshire county, United Kingdom, presented to the psychiatric inpatient department with complaints of insomnia and hearing alien voices for the last five days. He has no prior medical or family history of any psychiatric disorder. He had trouble sleeping for more than a week. Furthermore, for the past four days, he had been hearing voices telling him that he was being followed by the devil. Prior to these occurrences, he had no medical diagnosis of any illness. He denied consuming alcohol or other drugs, and he did not have any known drug allergies. Upon reviewing his recent medical history, it was discovered that the patient has been taking 500 mg of prophylactic chloroquine per week for the past four weeks, as he recently attended a business meeting in Guatemala. His primary care physician advised him to begin malaria prophylaxis in accordance with World Health Organization (WHO) recommendations, as he was traveling to a chloroquine-sensitive region. This was his first trip to a foreign country, and he had no prior history of malaria. After a week-long stay in Guatemala, he returned to his home country and continued his prophylaxis. However, during the fourth week of his prophylactic chloroquine treatment, he began to feel irritable and had difficulty sleeping at night. In addition, the onset of voices prompted him to visit the inpatient department. 

His physical examination and vital signs were unremarkable. No fever, rash, nausea, vomiting, or headache was present. He was agitated and was getting suspicious of the staff as being involved in a conspiracy against him. His speech was disorganized. He became repeatedly agitated and began to verbally abuse the staff. He even threw his shoes at a nurse, accusing her of being the devil. Consequently, intramuscular haloperidol was administered to treat this patient's acute psychomotor agitation. His Mini Mental Score was 28, which ruled out delirium as no alternating level of consciousness was found.

Numerous laboratory tests, including complete blood count (CBC), white blood cells (WBC), liver function tests (LFT), renal function test (RFT), thyroid function test (TSH), antinuclear antibodies (ANA), HIV, malaria card test, typhoid test, serum electrolytes, and urine drug tests were performed, but none of them revealed any abnormality. Based on the Diagnostic and Statistical Manual of Mental disorders, fifth edition (DSM-5), the patient was diagnosed with chloroquine-induced psychosis. Furthermore, the Naranjo Adverse Drug Reaction Probability Scale assessment revealed a score of 7 making chloroquine a probable cause of his psychotic symptoms (Table 1).

**Table 1:** Naranjo Adverse Drug Reaction Probability Scale

Questions	Yes	No	Do not know	Patient
1. Are there previous conclusive reports on this reaction?	1	0	0	1
2. Did the adverse event appear after the suspected drug was administered?	2	-1	0	2
3. Did the adverse reaction improve when the drug was discontinued or a specific antagonist was administered?	1	0	0	1
4. Did the adverse event reappear when the drug was re‐administered?	2	-1	0	0
5. Are there alternative causes (other than the drug) that could on their own have caused the reaction?	-1	2	0	2
6. Did the reaction reappear when a placebo was given?	-1	1	0	0
7. Was the drug detected in blood (or other fluids) in concentrations known to be toxic?	1	0	0	0
8. Was the reaction more severe when the dose was increased or less severe when the dose was decreased?	1	0	0	0
9. Did the patient have a similar reaction to the same or similar drugs in any previous exposure?	1	0	0	0
10. Was the adverse event confirmed by any objective evidence?	1	0	0	1
Total score :				7

His psychosis was not associated with any mood changes. Based on the clinical profile of the patient, a second-generation antipsychotic, quetiapine, starting with an initial dose of 25 mg once a day and titrating up to 300 mg twice a day, was prescribed. He was also given 0.5 mg of alprazolam at night.

His symptoms improved within a week of treatment, and he was discharged with counseling and instructions to follow up every two weeks. His medications were continued for three months and gradually tapered off. 

## Discussion

Malaria is a common infection that affects people all over the world, with the WHO reporting up to 229 million cases in 2019 [4]. To treat malaria, the WHO recommends artemisinin-based combination therapies (ACTs). They are considered the first and second lines of treatment because alternative antimalarial drugs such as chloroquine, doxycycline, and quinine have significantly more side effects [5]. Other options as approved by CDC guidelines include atovaquone-proguanil as prophylaxis in the military population. Additionally, doxycycline, chloroquine, and mefloquine are used as preventatives. Chemoprophylactic agents are recommended for visitors to malaria-endemic countries. In some cases, these chemoprophylactic anti-malarial drugs can have serious side effects. Mefloquine use has been linked to the occurrence of neuropsychiatric side effects such as anxiety, depression, insomnia, frank psychosis, and dizziness [4]. Similarly, studies on chloroquine, which has also been linked to psychosis in patients, have been conducted [6]. Since World War II, chloroquine has been preferred over other antimalarial drugs [7]. Its use has declined over time due to a narrow margin of safety and the rapid occurrence of toxicity. Overdoes of chloroquine carry a 10-35% risk of death. Cases of chloroquine-induced psychosis have been reported since 1958. The current literature reports symptoms of depersonalization, anxiety, derealization, and visual hallucination in chloroquine patients [3,7]. In the current century, the occurrence of psychosis induced by chloroquine has become rare, especially in children. One of the recent cases includes a nine-year-old boy who was admitted to the hospital with recurrent fever, abnormal behavior, and insomnia. He was given artesunate for three days, followed by two days of oral chloroquine. He reported hearing voices, increased sensitivity, eye irritation, sleep disruption, disorientation, and visual hallucinations. He was diagnosed with a medication-induced psychotic disorder and was treated for a week with olanzapine and clonazepam. This treatment plan relieved his symptoms in three days. This case shows the re-emergence of chloroquine-induced psychosis in both adults and children [2]. The first case report of chloroquine-induced psychosis in a coronavirus disease 2019 (COVID-19) infected patient was published in 2020. The patient suffered from visual hallucinations, mystical delusions, and severe anxiety. The Naranjo scale with a total score of 7 showed the chloroquine-induced psychosis as “probable.” The symptoms of psychosis disappeared after the discontinuation of chloroquine [8].

Another case report of a 34-year-old woman shows that she developed chloroquine-induced psychosis within two days of taking the drug. The symptoms persisted for four months because the patient refused to take antipsychotic medications to treat the condition. The longer half-life of chloroquine and other antimalarials causes prolonged psychiatric symptoms [7]. Antimalarials can cause general psychotic symptoms such as delusions, visual, auditory, and tactile hallucinations, suspiciousness, depression, anxiety, restlessness, and confusion. If such symptoms occur, it is recommended that prophylactic antimalarials be discontinued and a substitute drug be used [6]. The range of neuropsychiatric symptoms observed among 60 patients in a study by Bhatia and Malik is given in Table 2. The results show that psychosis was the most common adverse effect in the individuals followed by anxiety state and seizures. The symptoms were relieved after 2-21 days of discontinuation of chloroquine [9].

**Table 2:** Study showing common neuropsychiatric symptoms in patients who took chloroquine as a prophylactic for malaria. Source: Bhatia and Malik, 1994 [9]

Complication	Age (years)	Patient	Dosage (g)	Onset (days)
Organic Psychosis	6 to 36	32	1.0 to 2.4 (Mean 1.8)	2 to 4
Schizophrenia	13 to 35	12	1.25 to 2.0 (Mean 1.6)	3 to 6
Mania	10 to 16	4	1.5 and 2.2	5 to 7
Depression	18 to 30	4	1.5 and 2.4	4 to 7
Anxiety state	28 to 32	4	2.0	2 to 3
Seizures	12 to 26	4	1.0 and 2.0	3 to 4

The precise mechanism of action behind psychiatric symptoms caused by chloroquine is unknown. However, chloroquine can easily cross the blood-brain barrier and accumulate in the brain. This could explain the psychotic side effects [6]. It has been suggested that chloroquine causes the alteration in dopamine levels in the brain causing a hyperdopaminergic response. Another speculation is the inhibitory action of chloroquine on acetylcholinesterase [10]. Another possibility is that calcium homeostasis is disrupted. The pathophysiology of psychiatric symptoms caused by chloroquine is unknown. According to one study, the mechanism involves the inhibition of P-glycoprotein. It is a protein that is in charge of removing drugs from the central nervous system. Chloroquine binds to the P-glycoprotein, inhibiting its activity. This results in increased antimalarial concentrations in the brain [6]. A physical examination, family history, assessment of co-occurring problems, and psychosocial functioning can all be used to perform a comprehensive clinical assessment of a patient with new-onset psychosis. The DSM-5 diagnostic criteria are used to diagnose psychosis. The patient should have one or more psychiatric symptoms, such as hallucinations, delusions, disorganized/catatonic behavior, or disorganized speech. The episode should last at least a day, including the individual's return to normal functioning. Laboratory testing, neuroimaging, and a thorough physical examination should be performed if there is an underlying medical condition [11]. Second-generation antipsychotics such as quetiapine, risperidone, and olanzapine have been used to treat drug-induced psychosis, and their use is dependent on the symptoms, medication side effects, and patient comorbidities [2,6,11]. Risperidone is typically started at 1 mg twice daily, while aripiprazole is typically started at 10 mg twice daily. Short-acting benzodiazepines can be given to calm agitation and anxiety [11]. The dose can be titrated to the desired therapeutic range. Chloroquine, mefloquine, and other antimalarials should be used with caution and only after the underlying condition has been correctly diagnosed. While taking antimalarials, a patient should be educated about the side effects and be advised to seek help as soon as any of the unusual side effects appear [8].

## Conclusions

With the frequent use of anti-malarials in the past few decades, a multitude of adverse effects has been reported. Chloroquine and its use as an antimalarial prophylactic is well known to physicians worldwide. Even when used as a prophylactic, the potential neuropsychiatric side effects of chloroquine can not be overlooked. As a physician, one must understand the need of educating patients at the outset of prophylaxis, and the patient should be advised to seek help as soon as any of the unusual side effect appears.
